# Examining Daily Mental Health in Hispanic and Latino Dementia Caregivers Using an Intensive Longitudinal Design

**DOI:** 10.1093/geroni/igaf122.1702

**Published:** 2025-12-31

**Authors:** Natashia Bibriescas, Loreli Alvarez, Frank Puga

**Affiliations:** University of Alabama at Birmingham, Birmingham, Alabama, United States; The University of Alabama at Birmingham, Birmingham, Alabama, United States; University of Alabama at Birmingham, Birmingham, Alabama, United States

## Abstract

Caregiving for individuals with dementia is a dynamic experience characterized by daily variations in stress and mental health. However, most research relies on cross-sectional data, which may overlook these variations. This is especially true for Hispanic and Latino (H&L) caregivers, who experience disproportionate caregiving burden and stress. This study examined the effectiveness of recruitment strategies, the feasibility of repeated daily assessments, and mental health trajectories using data from the Nuestros Días (Our Days) Study. Participants (n = 180) completed daily diary surveys for 21 consecutive days at baseline, 6 months, and 12 months. The effectiveness of recruitment strategies was assessed using enrollment data, while feasibility was assessed using retention and completion data. Multilevel mixture modeling was used to identify distinct trajectories of depression and anxiety symptoms over time. Digital marketing was the most successful recruitment strategy, accounting for 76.5% of total enrollments. Retention rates were high, with 98.9% of expected participants completing wave one, 84.3% completing wave two, and 84.8% completing wave three. Preliminary mixture model analyses from wave one (n = 178) revealed distinct depression and anxiety symptom trajectories among H&L dementia caregivers. Three patterns emerged for both depression and anxiety symptoms: 1) caregivers with high symptoms that decreased over time, 2) caregivers with high symptoms that increased over time, and 3) caregivers with consistently low symptoms. These findings underscore the heterogeneity in H&L caregiver mental health over time and highlight both the feasibility of intensive daily dairy data collection and its value in capturing the dynamic nature of caregiving experiences.

